# Hip Abductor Muscle Fatigue Induces Different Strategies During Disrupted Postural Control

**DOI:** 10.3389/fspor.2022.918402

**Published:** 2022-06-29

**Authors:** Jeanne Dury, Gilles Ravier, Fabrice Michel

**Affiliations:** ^1^Université de Franche Comté, Laboratoire C3S (EA 4660), UFR STAPS, Besançon, France; ^2^Laboratoire Athlète Matériel Environnement, Besançon, France; ^3^Université de Franche Comté, Laboratoire Nanomédecine (EA 4662), Besançon, France; ^4^Service de Médecine Physique et de Réadaptation, CHRU Hôpital Jean Minjoz, Besançon, France

**Keywords:** balance, ankle, destabilization, electromyography, inertial measurement

## Abstract

**Background:**

Ankle sprain is one of the most common injuries in sport, and hip abductor muscle weakness has recently been reported as a predisposing factor. Currently, the influence of hip abductor muscle fatigue on ankle joint control has not been elucidated during an ankle disturbed balance exercise. This study aimed to determine the influence of hip abductor muscle fatigue on ankle joint control during a disturbed balance task, and to consider inter-individual variability in the kinematic and neuromuscular reorganizations implemented.

**Methods:**

Twenty-six healthy subjects (13 males; 13 females) performed a unipedal postural balance task with eyes closed before and after a fatiguing exercise (up to a 50% decrease in strength) of the hip abductor muscles. Subjects completed balance task while equipped with an ankle destabilization device that allows inversion/eversion movements. Electromyographic (EMG) activity of the gastrocnemius lateralis (GastL), peroneus longus (PL) and brevis, tibialis anterior, and gluteus medius were recorded during task. Kinematics (e.g., frontal foot angulation) of the ankle complex were determined using inertial measurement units.

**Results:**

In the overall group, no significant time, sex or interaction effect was observed for kinematic and EMG variables. However, when considering individual responses to hip fatigue, 14 subjects decreased the standard deviation of frontal angulation (−30%) suggesting enhancement of ankle joint control, while 12 subjects increased it (+46%). Normalized EMG for PL and GastL muscles changed with fatigue for both these groups. However, variations were significantly different between groups (*p* = 0.027 for PL and *p* = 0.006 for GastL). Indeed, the contribution of ankle muscles increased for the enhanced-stability group while no change for the impaired-stability group.

**Conclusion:**

These results highlight that subject adopt different neuromuscular and kinematic ankle strategies to control ankle destabilization in response to hip abductor muscle fatigue. Frontal foot angulation variability seemed to be a valuable marker to detect the type of strategy employed. The strategy adopted by the impaired-stability group might have important implications when analyzing risk factors for ankle sprains. Further studies should consider individual responses to fatigue, to understand which factor could predispose athletes to use of one or other strategy.

## Introduction

Ankle sprains represent the most common musculoskeletal injury in sports (Fong et al., 2007; Waterman et al., 2010) and have a high rate of re-injury, ranging between 12 and 47% according to the sport specificity (Herzog et al., 2019). It is well-established that impairment of postural control is one of the most important risk factors (Delahunt and Remus, 2019). Indeed, balance depends on sensorimotor integration capacities and is considered as a determinant factor in motor skills (Peterka, 2002).

Postural control is based on two different organizations involving both the ankle and hip joints, usually with predominance of either the ankle or the hip (Nashner and McCollum, 1985). The ankle strategy implies medio-lateral and antero-posterior modulations around the ankle and is often described as an inverted pendulum. The hip strategy results from trunk movements around the hip to regulate posture. The hip and ankle are also the most important joints involved in postural control. The strategy adopted by individual subjects can be influenced by deficiencies (musculoskeletal disorders, pathology, neuromuscular weakness). For instance, subjects with chronic low back pain are characterized by reduced dependence on the hip to maintain a static postural task (Mok et al., 2004). Moreover, subjects with distal deficits, such as chronic ankle instability, showed an alteration of hip/ankle coordination during walking (Yen et al., 2017). These pathologies lead to an impairment of balance, particularly on the medio-lateral axis.

Furthermore, the strategy adopted to maintain balance can also be affected by fatigue (Ritzmann et al., 2016). Gribble and Hertel (2004) showed an impairment of postural control in response to hip abductor/adductor fatigue. More recently, an increase in mean medio–lateral center of pressure displacement following exercise-induced fatigue of hip abductors was reported (Lee and Powers, 2013). Furthermore, with diminished hip abductor strength, a shift toward an ankle strategy to maintain unipedal balance was demonstrated (Lee and Powers, 2014). Moreover, changes in ankle kinematic and electromyographic activity during landing tasks have been reported in response to hip abductor muscle fatigue (Lee and Powers, 2013; Gafner et al., 2018). Accordingly, less plantarflexion and greater activity of the peroneus longus and tibialis anterior were demonstrated with hip abductor fatigue during landing. Based on these studies, it seems established that hip abductor fatigue impairs postural stability and impacts ankle response during landing. Nevertheless, high inter-individual variability was reported in response to fatigue (Bergstrom et al., 2020), which might reflect multiple individual specific motor reorganizations (Bonnard et al., 1994; Kennedy et al., 2012). Thus, the influence of fatigue could be dependent on the motor reorganization employed by each individual. Therefore, it would be interesting to determine whether hip abductor fatigue can lead to various compensatory responses among subjects during postural control.

Traditionally, assessment of postural stability uses a force plate to analyze center of pressure displacements (Panzer et al., 1995; Nakamura et al., 2001). Several parameters have been used to describe postural stability, such as the standard deviation around the mean position of the center of pressure. While this biomechanical variable reflects the whole-body organization, it seems limited as a means to provide information on the specific adaptation of the ankle joint. In the context of ankle sprain prevention and chronic ankle instability, a device equipped with an inertial measurement unit (IMU) implying a specific rear-foot destabilization has been shown to be a relevant device in ankle investigation (Terrier et al., 2017). In order to evaluate the influence of hip muscle fatigue specifically on ankle joint control, this device seems well-adapted. In recent decades, IMU have become increasingly popular thanks to their low cost and their ability to record kinematic variables during functional movement. Indeed, IMUs record accelerations and angular velocities in 3 planes, enabling assessment of joint kinematics, or gait and posture biomechanics (Washabaugh et al., 2017; Weygers et al., 2020). Accordingly, the position of each of the three planes can be derived from the IMU and the standard deviation of the frontal position may represent a valuable indicator of ankle joint mobility.

The strategy used to maintain balance can be influence by different factors as pathology and fatigue. Indeed, hip abductor fatigue seems to modulate the postural strategy toward an ankle strategy. Nevertheless, muscular fatigue induces high inter-individual variability in motor reorganization, although the variability in response following hip abductor fatigue is not clearly established.

Therefore, the objective of the study was to determine the influence of hip abductor muscle fatigue on i) medio-lateral ankle joint control during a specifically disturbed balance task; and ii) individual reorganization strategies adopted. We hypothesized that hip abductor fatigue led to an impairment of medio-lateral ankle joint control. Furthermore, we expected a high inter-individual variability in neuromuscular response following fatigue that could characterize different reorganization profiles.

## Materials and Methods

### Population

Sample size was estimated *a priori* using G^*^Power 3.1 software. Considering a mixed 2 × 2 ANOVA approach a minimum of 24 individuals was required (α error = 0.05; power β = 0.80; Cohen's *f* = 0.30). In order to take account of the missing data, it was decided to include a total of 26 subjects. Twenty-six subjects, 13 males (21.6 ± 1.9 years; 71.0 ± 8.3 kg; 176.4 ± 5.4 cm) and 13 females (21.3 ± 1.8 years; 58.8 ± 6.5 kg; 165.6 ± 5.3 cm) participated in this study. All participants were free of lower limb injuries in the past 6 months and did not present functional ankle instability which was tested by the Cumberland Ankle Instability Tool (CAIT). Participants had to complete the CAIT before inclusion and only subjects with a CAIT score > 24 were selected, thus excluding patients with chronic ankle instability. All participants provided written informed consent. The study was conducted according to the Declaration of Helsinki and was approved by the Ethics Committee CPP Sud Est V under the number 2021-A02759-32 (CPP Sud Est V, CHU Grenoble, France).

### Procedure

**Figure 1:**
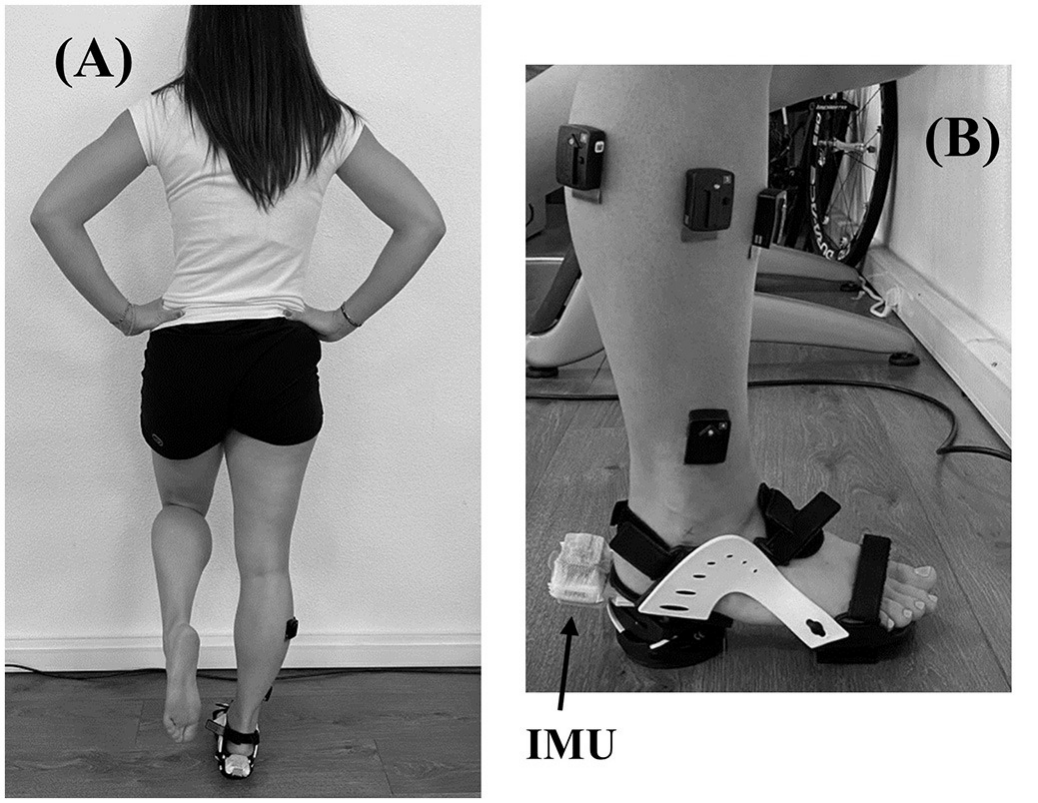
**(A)** Postural test execution; **(B)** Destabilization device.

Subjects performed two sessions. The first was a familiarization session, aimed at explaining the tests and the measurement procedures, testing the balance task, and collecting personal information about age, weight, height, and dominant limb (determine as the preferred push-off leg during unipedal jump). The second session was completed within 1 week after familiarization (3.5 ± 1.8 days). The second session was the experimental session. During this experimental session, participants performed a postural balance test before and immediately after a fatiguing exercise of the hip abductor muscles. The postural test (Figure 1A) consisted of maintaining balance on the dominant leg with the eyes closed and wearing the ankle destabilization device for 20 s. One trial was performed in pre fatigue and post fatigue. The trial was repeat if subjects lost balance or opened eyes for few seconds. A training time was process before recording until subjects were confident and focused on the test. The destabilization device, shown in Figure 1B (Myolux Medik II, ICC physio, Le Bourget du Lac, France), was a sandal composed of an articulator under the rear foot, allowing destabilization around the Henke axis ranging between −15 and 15 degrees of eversion/inversion. Subjects performed the test with the knee extended and hands placed on the hips, restricting compensations.

**Figure 2:**
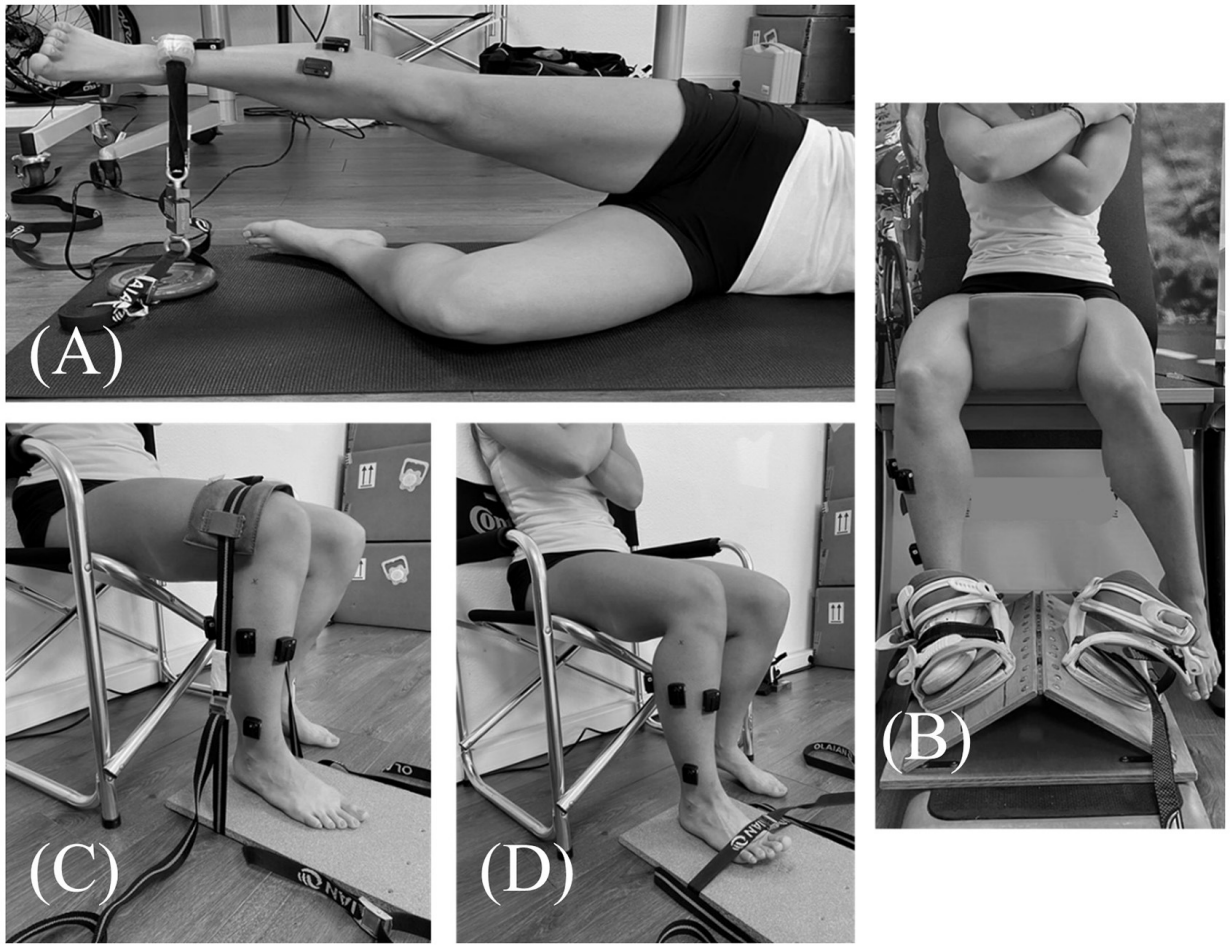
Maximal voluntary isometric contraction of **(A)** Gluteus medius, **(B)** Peroneus longus and brevis, **(C)** Gastrocnemius lateralis, **(D)** Tibialis anterior.

Fatiguing exercise of hip abductor muscles was based on previous studies (Gafner et al., 2018). Subjects were positioned lying on their side and had to repeat hip abduction movements of 30° at a rate of 60 repetitions per minute. When the subjects were no longer able to maintain range of motion or cadence the round was stopped. After each round, participants reported perceived exertion on Borg's CR10 scale, and a measurement of isometric hip abductor muscle strength was performed (Figure 2A). Subjects restarted another round until they had a decrease in hip abductor strength of 50% maximal voluntary isometric contraction (MVIC) compared to the pre-fatigue strength assessment. During the strength measurements, subjects were encouraged to push up as fast and as strong as possible under the strap fixed on the floor. Three trials were performed before the fatiguing exercise to ensure reproducibility of measure and an understanding of instructions by subjects. Then, the best performance strength was retained for further analysis. Between rounds of fatigue, only one measurement of strength was performed to avoid a discontinuity in the fatiguing exercise. The post-fatigue balance task was performed immediately after the fatigue session to limit recovery (<1 min).

### Materials and Measurements

#### Surface Electromyography (EMG)

During the postural test, muscle activity of the gluteus medius (GlutM), peroneus longus and brevis (PL, PB), tibialis anterior (TA) and gastrocnemius lateralis (GastL) were recorded using a surface electromyography device (Delsys trigno^TM^, Delsys Inc., Natick, USA). EMG data were collected at 1,111 Hz, and band pass filter of 20–450 Hz. Data collection was done using EMGworks^®^ Acquisition software (Delsys Inc., Natick, USA). According to the SENIAM recommendations, the skin was shaved, abrased and cleaned prior to positioning the electrodes. Furthermore, MVIC of each muscle were performed at the beginning of the experimental procedure in order to normalize EMG data according to this reference. The positions during MVIC of GlutM, PL/PB, GastL, and TA were illustrated in Figures 2A–D, respectively. For each muscle, participants were instructed to produce three contraction trials against resistance as hard and as fast as possible and to hold the contraction for 4 s. A rest period of 1 min was respected between trials. All EMG signals were smoothed with a 20 sample window root-mean-square (RMS) to maintain the relationship with kinematic variables. EMG MVIC signals were smoothed with a 200 ms mean sliding window. The maximum value of these processed signals was selected for each muscle. Then, these values served as a reference to normalize EMG signals recorded during test. Signal recordings during the postural test were averaged over a 10 second period in the middle of the test (from 5 to 15 s of test), considered as the most stable period of the test, and then expressed as a percentage of MVIC.

#### Inertial Measurement Unit (IMU)

An IMU (Delsys trigno^TM^, Delsys Inc., Natick, USA) was fixed on the destabilization device during the postural test in order to record accelerations in three-dimensional axes (148 Hz). The accelerations were low pass filtered with a cut-off frequency of 5 Hz. Frontal angulation of the foot-ankle complex (°) corresponding to the range of pronation/supination was calculated using accelerations of the sagittal (Ax), frontal (Ay), and transverse (Az) planes using the following equation, and was then converted from radians to degrees:


$$\text{Frontal angulation }=tan^{-1}(Ay/\sqrt{Ax^{2}+Az^{2}}),$$

where tan^−1^ correspond to the arctan function.

Based on frontal angulation, the mean and the standard deviation (SD) was defined during the 10 second period selected for EMG signal processing.

#### Hip Abductor Strength

Hip abductor strength was assessed using an S-shaped force transducer (PCE-FB 2K, PCE Instruments^®^, Meschede, Germany) sampled at 40 Hz with a range of measure of 0.5 N (50 g) to 2,000 N (200 kg) with a full-scale accuracy of ±0.1% and collected with PCE software. Hip abductor strength was determined for pre- and post-fatiguing exercise from the maximal strength (N) measured with 1 s sliding mean windows using a custom-built MATLAB routine (version R2019b, The MathWorks, MA, USA).

### Statistical Analysis

Statistical analysis was performed using JASP (version 0.13.1.0). Data were checked for normality and homogeneity using the Shapiro–Wilk and Levene tests, respectively. For non-normal sample distributions, a logarithmic transformation was performed (log10).

For each index (SD of frontal angulation, mean of frontal angulation, GlutM, PL, PB, TA, GastL), a mixed 2 × 2 ANOVA was performed on the whole group with time (pre-, post-fatiguing exercise) and sex factors.

Normalized changes occurring with fatigue were determined for each index according to the following equation:


$$\text{Normalized change }(\%)\text{ = }(\frac{\text{post – pre}}{\text{pre}}\times \text{100})\;$$

Independent sample *t*-tests or Mann–Whitney tests were used, depending on the results of the Shapiro–Wilk and Levene tests, to compare normalized changes (%) of kinematic and neuromuscular variables between the two groups. The significance level was set at 0.05 for these comparisons.

Furthermore, paired sample *t*-tests or Wilcoxon tests, according to the normality assessment, were performed to compare raw values evolution of kinematic and neuromuscular variables with fatigue for each group. Independent sample *t*-test or Mann–Whitney tests were also used to compared raw values difference between groups in pre- and post-fatigue. Considering the multiple comparisons performed, a Bonferroni correction was applied for these pairwise comparison. Thus, the significance level was set at 0.01 (0.05/4).

Furthermore, effect size (d) was calculated using Cohen's d and was interpreted as large (≥0.80), moderate (0.50–0.79), small (0.20–0.49), or trivial (<0.20) (Cohen, 1988).

## Results

### Overall Population

Mixed ANOVA performed for each neuromuscular and kinematic parameter did not reveal any statistically significant differences between pre- and post-fatigue conditions, male and female groups, or interactions. The evolutions of each index with fatigue in the whole population were +2.07% MVIC (Confidence Interval 95%: −0.69; 5.03) for GlutM; +1.51% MVIC (−5.91; 8.54) for PL; +2.19% MVIC (−2.64; 7.02) for PB; −0.91% MVIC (−3.06; 1.25) for TA; −0.22% MVIC (−2.79; 2.34) for GastL; −0.29° (−0.77; 0.20) for SD of frontal angulation; −1.06° (−0.40; 2.51) for mean of frontal angulation.

### Inter-group Comparisons

Based on the change in the frontal ankle mobility index (i.e., the standard deviation of the ankle frontal position from IMU) with fatigue, participants were classed in two groups after the fatiguing exercise, namely: the enhanced-stability group, defined as participants showing a decrease in frontal ankle mobility index [14 subjects (7 females, 7 males)]; and the impaired-stability group, defined as participants showing an increase in frontal ankle mobility index [12 subjects (6 females and 6 males)].

#### Group Comparisons of Kinematic Variables

**Figure 3:**
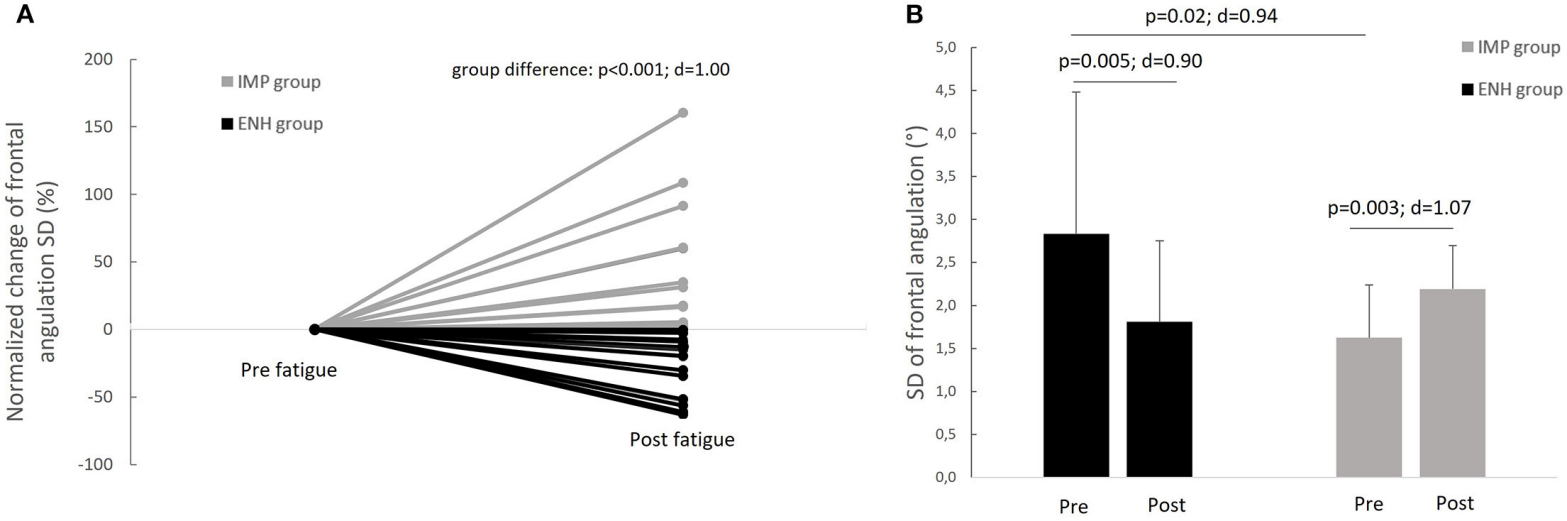
**(A)** Inter-individual variability of the evolution of frontal angulation standard deviation index in response to fatigue and **(B)** comparison of the evolution of frontal angulation standard deviation index with fatigue between the enhanced-stability (ENH) and the impaired-stability (IMP) groups.

The inter-individual variability of SD of frontal angulation with fatigue is displayed on the Figure 3A. The enhanced-stability group presented a significant decrease in frontal angulation SD of 1.02 ± 1.14 (CI 95% 0.37–1.68) degrees (Figure 3B) corresponding to a normalized change of −30%, whereas the impaired-stability group presented a significant increase in frontal angulation SD of 0.57 ± 0.53 (CI 95% 0.23–0.90) degrees (Figure 3B), corresponding to a normalized change of +46%. Considering the Bonferroni correction, the enhanced-stability group tended to present a higher SD of frontal angulation than the impaired-stability group with a large effect in the pre-fatigue state (Figure 3B). The Mann–Whitney test confirmed a significant difference in normalized change of frontal angulation SD between groups (Figure 3A). Considering the mean of frontal angulation, both groups presented no significant evolution of this index with fatigue (impaired-stability group: *p* = 0.85, *d* = 0.06; enhanced-stability group: *p* = 0.06, *d* = 0.55). Furthermore, there was no significant difference between groups in pre and in post-fatigue (pre-fatigue: *p* = 0.51, *d* = 0.26; post-fatigue: *p* = 0.35, *d* = 0.18). The comparison of normalized changes between groups (−89% for impaired-stability group and −10% for enhanced-stability group) revealed no significant difference (*p* = 0.37, *d* = 0.36).

#### Group Comparisons of Hip Abductor Strength

Both groups presented a significant decrease in hip abductor strength compared to baseline. The enhanced-stability group presented a significant decrease in force of 70.05 ± 22.3 N (*p* < 0.001) and the impaired-stability group a significant decrease of 80.25 ± 22.6 N (*p* < 0.001). However, the Mann–Whitney test revealed no statistically significant difference between groups in the evolution of force (normalized change) with fatigue (*p* = 0.25).

#### Group Comparisons of Muscle Activity

**Figure 4:**
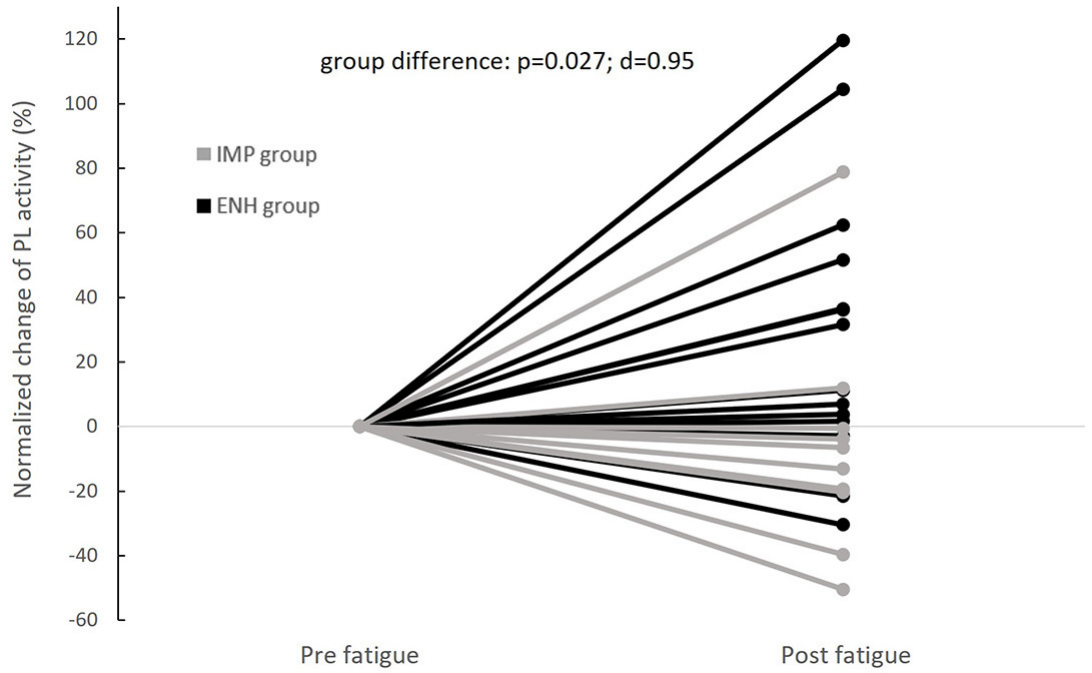
Individual evolutions of normalized change in Peroneus Longus (PL) activity with fatigue between groups (IMP, impaired-stability group; ENH, enhanced-stability group).

**Figure 5:**
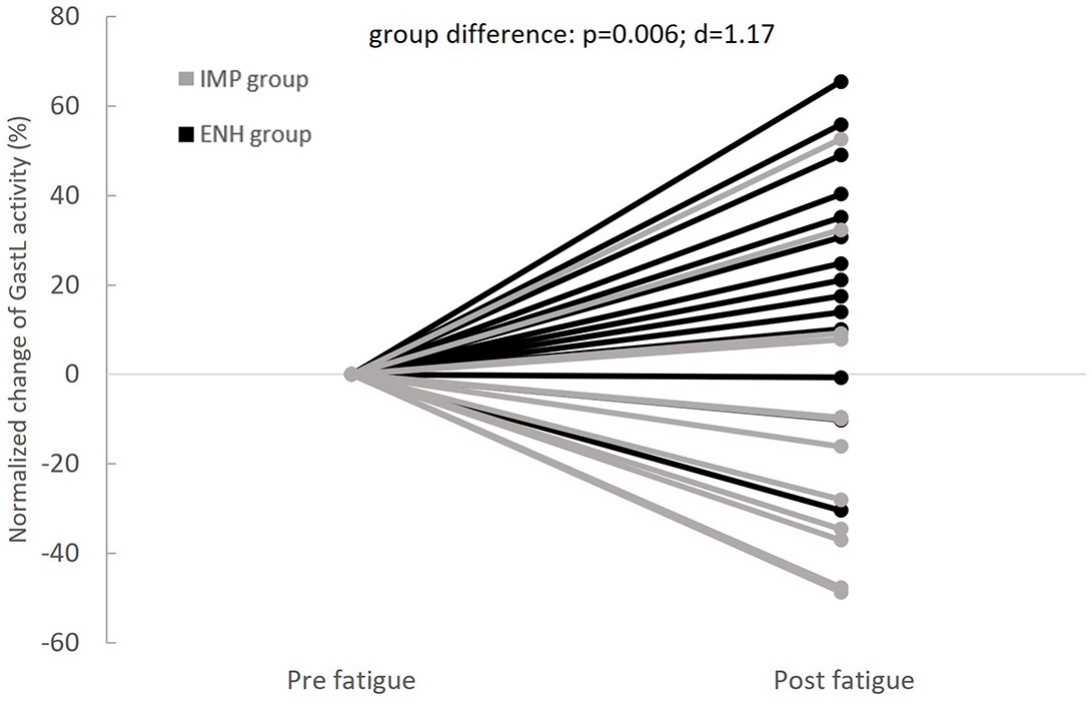
Individual evolutions of normalized change in Gastrocnemius Lateralis (GastL) activity with fatigue between groups (IMP, impaired-stability group; ENH, enhanced-stability group).

Normalized changes in PL and GastL activity appeared significantly different between groups. These results are displayed in Figures 4, 5, respectively. The enhanced-stability group tended to increase PL activity of +31% (*p* = 0.06; *d* = 0.52) and GastL activity of +23% (*p* = 0.03; *d* = 0.65) with a moderate effect suggesting clinical relevance, while the impaired-stability group had a normalized change decrease in the activity of these muscles of −6 and −11%, respectively, without a significant evolution. Considering the Bonferroni correction, PL activity tended to be higher in the enhanced-stability group in post fatigue than impaired-stability group with a large effect (*p* = 0.03; *d* = 0.94). For GlutM, TA and PB, the normalized changes did not differ significantly between groups.

## Discussion

The purpose of this study was to determine the influence of hip abductor muscle fatigue on (i) medio-lateral ankle joint control during a specifically disturbed balance task; and (ii) individual reorganization strategies adopted.

Our study did not reveal any impairments in medio-lateral ankle joint kinematic in response to fatiguing exercise, or any specific neuromuscular compensations in the overall group. These results are at odds with previous studies, which highlighted perturbed unipedal stability with fatigue (Paillard, 2012), particularly hip abductor fatigue (Gribble and Hertel, 2004). Our study was based on analysis of the deviation of the ankle position in the frontal plane from IMU, whereas previous studies focused on center of pressure displacement and velocity from a force plate. The force plate measurement reflects whole-body organization, but not specifically that of the ankle, which could explain the discrepancy with our results. Furthermore, a modulation of ankle neuromuscular response has previously been described in response to hip abductor fatigue during landing (Gafner et al., 2018). During the disturbed balance task, we did not observe any neuromuscular evolution of the ankle following fatigue in the overall group. The difference between the task performed in our study (i.e., a balance task with a destabilizing device) and in the previous study (i.e., a landing task) could explain these inconsistent results. Moreover, during postural control, it appeared more difficult to counteract proximal fatigue effects (hip, knee) than distal fatigue (ankle). According to the literature, the shift from the hip toward an ankle strategy may not be possible in postural tasks (Paillard, 2012). Finally, both neuromuscular and kinematic response with fatigue appeared highly variable between subjects when considering the whole experimental group (Figure 3A), which precludes drawing any definitive conclusions from our overall study population.

In order to take account of this high inter-individual variability, two groups were constituted according to the different changes in ankle mobility in response to fatigue. Half of the participants presented an increase in the medio-lateral ankle mobility index (SD of frontal angulation), while the other half had a decrease (Figure 3A). These findings suggest that hip abductor muscle fatigue could affect athletes differently. In a previous study, a similar segmentation of the population was performed, and revealed two postural consequences of mechanical ankle constraint, whereby some subjects increased postural sway with ankle constraint, while others decreased it (Tsai et al., 2018). These different behaviors were explained by authors of that study as an individual adaptive strategy consisting in either favoring hip motion (or not) to compensate for the ankle constraint.

The different changes of ankle mobility showed in our study (+46% for impaired-stability group and −30% for enhanced-stability group) might be related to the neuromuscular strategies adopted by each individual. Indeed, the neuromuscular compensations employed by the individuals in our study appeared to differ between the impaired-stability and enhanced-stability groups (Figures 4, 5). The enhanced-stability group increase PL and GastL activity, whereas the impaired-stability group seemed not to modify it. In the literature, it has previously been shown that the neuromuscular reorganization implemented with fatigue can vary from one individual to another (Paillard, 2012). In fact, the redundancy of the neuromuscular system implies various possibilities to compensate for localized fatigue during multi-joint tasks such as posture (Côté et al., 2002). Two strategies were also previously shown in the literature, which aimed at either increasing the activity of muscles affected by exercise-induced fatigue, or involving muscles of other joints (Bonnard et al., 1994). The involvement of the same muscles as the fatiguing joint leads to an increase in muscular anticipation and activation, while compensation with other joints implies activation of muscles that were inactive in the pre-fatigue state (Bonnard et al., 1994; Strang et al., 2009). According to our results, individuals in the enhanced-stability group tend to reinforce the dependency of the ankle joint to perform the specifically disturbed balance task with proximal fatigue. Considering the change in ankle kinematics, this strategy leads to decrease ankle mobility index, despite the fatigue. However, individuals in the impaired-stability group appeared unable to implement the same strategy. This group could compensate for fatigue by using other joints to further stabilize proximal joints, which appears to be less effective in this specific destabilizing ankle task. Nevertheless, the impaired-stability group did not increase the gluteus medius activity with fatigue, which does not lead to the conclusion that they compensate with proximal muscles. Thus, it would be relevant to investigate other muscular groups (trunk and knee muscles) from proximal joints to better understand this strategy.

Moreover, the strategy adopted to compensate for fatigue could be related to the ability to reweight proprioceptive information, depending on the relevance and availability of sensory cues in the environment (Vuillerme et al., 2006). In healthy subjects, a high inter-individual variability in proprioceptive strategies used to maintain balance was shown by a previous study (Picot et al., 2022). This previous study highlighted two different proprioceptive behaviors, namely a rigid one, which results in maintenance of the strategy used despite the constraints; and a plastic behavior, which involved a shift of proprioceptive reliance in response to external constraints. In our population, it can be hypothesized that the impaired-stability group failed to appropriately reweight proprioceptive information according to the fatiguing event. This rigid behavior could explain the deterioration of frontal ankle mobility and the lack of ankle neuromuscular compensations. On the contrary, in response to hip abductor muscle fatigue, the enhanced-stability group increase ankle muscular contribution, they seem to have reinforce the ankle proprioceptive strategy that results in a decrease in ankle mobility index.

Nevertheless, our study has some limitations, notably the *a posteriori* dichotomization of the population. In fact, there were too few participants to consider more than 2 groups. For instance, it would seem interesting to add a third group which was not really impacted (in terms of mobility) by fatigue. Furthermore, the detection of vestibular impairments might help to better understand different strategies, particularly in the experimental task performed with eyes closed. Further studies are needed to understand specific strategies employed with fatigue. Because impaired-stability group do not present a significant decrease in ankle muscles activity in response to fatiguing task, it would be interesting to investigate the relationship between increment of SD of frontal angulation and risk factors of injuries.

## Conclusion

The results of the present study highlight that subject adopt different neuromuscular and kinematic ankle strategies to control ankle destabilization in response to hip abductor muscle fatigue. The frontal foot angulation variability seems to be a valuable marker to detect these neuromuscular strategies. Indeed, differences in change in mobility between the two groups were supported by different muscle reorganizations of the PL and GastL. The strategy adopted by the impaired-stability group might have important implications when analyzing risk factors for ankle sprains. Further studies should consider individual responses to fatigue in assessing which factor(s) could predispose athletes to adopting a specific strategy.

## Data Availability Statement

The raw data supporting the conclusions of this article will be made available by the authors, without undue reservation.

## Ethics Statement

The studies involving human participants were reviewed and approved by Ethics Committee CPP Sud Est V (CHU Grenoble, France) under the number 2021-A02759-32. The patients/participants provided their written informed consent to participate in this study.

## Author Contributions

JD collected and analyzed the data. JD and GR wrote manuscript. JD, GR, and FM designed the study and revised the manuscript. All authors contributed to the article and approved the submitted version.

## Conflict of Interest

The authors declare that the research was conducted in the absence of any commercial or financial relationships that could be construed as a potential conflict of interest.

## Publisher's Note

All claims expressed in this article are solely those of the authors and do not necessarily represent those of their affiliated organizations, or those of the publisher, the editors and the reviewers. Any product that may be evaluated in this article, or claim that may be made by its manufacturer, is not guaranteed or endorsed by the publisher.
